# Supplementary material for: Drug Repurposing using consilience of Knowledge Graph Completion methods
Source: bioRxiv. 2024 Aug 10:2023.05.12.540594. Preprint. [Version 3] doi: 10.1101/2023.05.12.540594 (PMC11326126; doi:10.1101/2023.05.12.540594)
Supplement: Supplement 1 [file media-1.pdf]

Supporting Information

S1 Fig. Describes the composition of nodes and edges in the MIND dataset as a whole. Upper: Heatmap of node type to node type counts. The y-axis and the x-axis correspond to the head and tail node respectively. Lower: Heatmap of Node type (both head and tail) to relation type counts; Heatmap colors are in exponential scale where darker signifies more and lighter less, respectively. Parentheses highlight the node type and relation type counts.

S1 Table. Scoring Functions for Knowledge Graph Embedding Algorithms.

Algorithm	Score Function
TransE	$-\|\|n_1 + r - n_2\|\|$
DistMult	$\langle n_1, r, n_2 \rangle$
ComplEx	$Re\langle n_1, r, n_2 \rangle$
RotatE	$-\|\|n_1 \odot r - n_2\|\|$

The angle brackets in DistMult and ComplEx denote a trilinear dot product. denotes the Hadamard product (element wise product) between the n1 and r embeddings. In brief, TransE, short for Translation Embedding, was developed in 2013 by Bordes et al., aimed to model relationships as translations in embedding space; where n2 should be close to the embedding of h when added to a vector, r [**?**]. DistMult, modeled relationships using a bilinear diagonal matrix and trilinear dot product as a scoring function [**?**]. ComplEx modified the DistMult model by embedding additional complex vector components but maintained the trilinear dot product as a scoring function [**?**]. Finally, RotatE modeled each relation as an element-wise rotation in complex vector spaces [**?**].

S1 File. Curation result of the Union ensemble approach. 25 randomly selected non-indication predictions were manually curated through a literature search for the union ensemble approach and each of its parts. Each compound was classified as having a ”Positive”, ”Neutral”, or ”Negative” effect on the predicted disease based on criteria outlined in the methods. In total, 200 predictions were made. The results are summarized in Table **??**.

S2 Table. KGE approaches Optuna optimization hyperparameter results.

Algorithm	Optimized MRR	Batch Size	Hidden Dimension Size	Negative Sample Size	Learning rate
TransE	0.054154	248	225	124	0.002098
DistMult	0.021927	212	300	88	0.000816
ComplEx	0.032595	252	250	84	0.000292
RotatE	0.068594	240	100	96	0.002329

S3 Table. CBR approaches Optuna optimization hyperparameter results.

Algorithm	Optimized MRR	Max Num Programs	Max Path Length	Max Branch	K Adj	Number of Paths Collected	Linkage
CBR	0.09672434757569749	25	3	100	10	1000	0
probCBR	0.20301439879421163	1000	3	100	5	1000	0

S4 Table. Kruskal-Wallis Test statistic and p-values of various ensemble lengths for each ensemble method.

Ensemble group	Indication group	statistic	p-value
Intersection	Positive & Negative	281752298.0	0.0
Intersection	Positive	134.8568	2.2155e-27
Intersection	Negative	3465.5996	0.0
Union	Positive & Negative	1351570.3371	0.0
Union	Positive	3210.1153	0.0
Union	Negative	1053174.30321	0.0

We report the Kruskal-Wallis test statistic value as well as the corresponding p-value for three groups: Positive, Negative, and Both. The Positive, Negative, and Both groups conduct the Kruskal-Wallis test on algorithm lengths 2 - 7 and only on approved indications, non-indications, and both approved and non-indications, respectively. Kruskal-Wallis H-Test compares the median of non-parametric distributions to identify if there is a statistically significant difference between the provided distributions. A low p-value suggests that at least one of the distributions is different from the others. .

S5 Table. Mann-Whitney U-Test statistic and p-values of various ensemble lengths for each ensemble method.

	Intersection		Union	
length	statistic	p-value	statistic	p-value
2	24670494.5	0.0	13638459952.0	0.0
3	281752298.0	0.0	145208921890.5	0.0
4	102138952.5	0.0	262928904076.0	0.0
5	13011720.0	0.0	128163422278.0	0.0
6	524477.5	3.5930e-127	17175072890.5	0.0
7	5063.0	5.2950e-17	407357083.0	0.0

We report the Mann-Whitney U-Test statistic value as well as the corresponding p-value for all six pairs of positive (approved indications) and negative (non-indications) distributions for both the intersection and union ensemble approaches. Mann-Whitney U-Test is a non-parametric method to compare whether two distributions are the same by randomly selecting X and Y from each distribution and measuring if the probability of $X > Y$ is the same as $Y > X$. A low p-value suggests that the probability of $X > Y$ is not the same as the probability of $Y > X$.

S6 Table. Sotalol hydrochloride's respective predicted rank for each link prediction method.

Algorithm	Hypertension Rank
CBR	3
probCBR	3
Rephetio	1
TransE	28
DistMult	86
ComplEx	27
RotatE	1
